# Crystal structure of 1-(2-chloro­acet­yl)-2,6-bis­(4-fluoro­phen­yl)-3,3-di­methyl­piperidin-4-one

**DOI:** 10.1107/S1600536814021278

**Published:** 2014-09-30

**Authors:** S. Jothivel, Jibon Kotoky, S. Kabilan

**Affiliations:** a Department of Chemistry, Annamalai University, Annamalainagar 608 002, Chidambaram, Tamil Nadu, India; b Division of Life Sciences, Central Instrumentation Facility, Institute of Advanced Study in Science & Technology (IASST), Guwahati 781 035, Assam, India

**Keywords:** Piperidone, weak hydrogen bonds, crystal structure

## Abstract

In the title mol­ecule, C_21_H_20_ClF_2_NO_2_, the piperidine ring adopts a slightly distorted boat conformation. The two benzene rings form a dihedral angle of 87.43 (1)°. A weak intra­molecular C—H⋯π inter­action is observed. In the crystal, weak C—H⋯O hydrogen bonds and weak C—H⋯π inter­actions connect the mol­ecules, forming a three-dimensional network.

## Abstract 2

In the title mol­ecule, C_21_H_20_ClF_2_NO_2_, the piperidine ring adopts a slightly-distorted boat conformation, the two benzene rings form a dihedral angle of 87.43 (1)° and a weak intra­molecular C—H⋯π inter­action is observed. In the crystal, weak C—H⋯O hydrogen bonds and weak C—H⋯π inter­actions connect mol­ecules forming a three-dimensional network.

## Chemical context   

Piperidones are an important group of heterocyclic compounds in the field of medicinal chemistry due to their biological activities, which include cytotoxic properties (Dimmock *et al.*, 2001 ▶). They are also reported to possess analgesic, anti-inflammatory, central nervous system (CNS), local anaesthetic, anti­cancer and anti­microbial activities (Perumal *et al.*, 2001 ▶). The present investigation was undertaken to establish the mol­ecular structure, the conformation of the heterocyclic ring and the orientation of the 4-fluoro­phenyl groups with respect to each other.
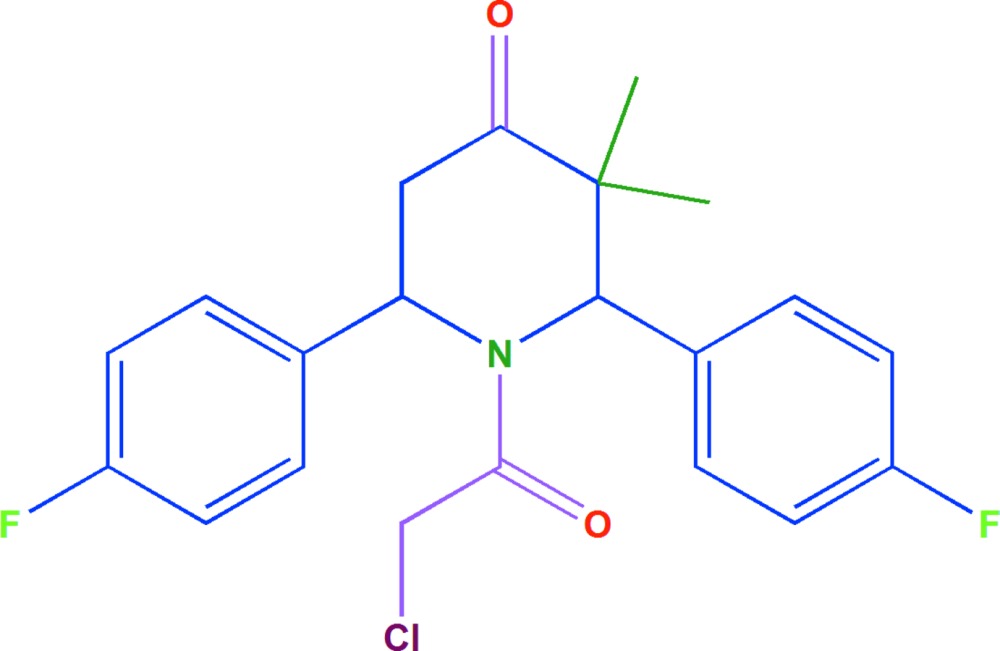


## Structural commentary   

**Figure 1:**
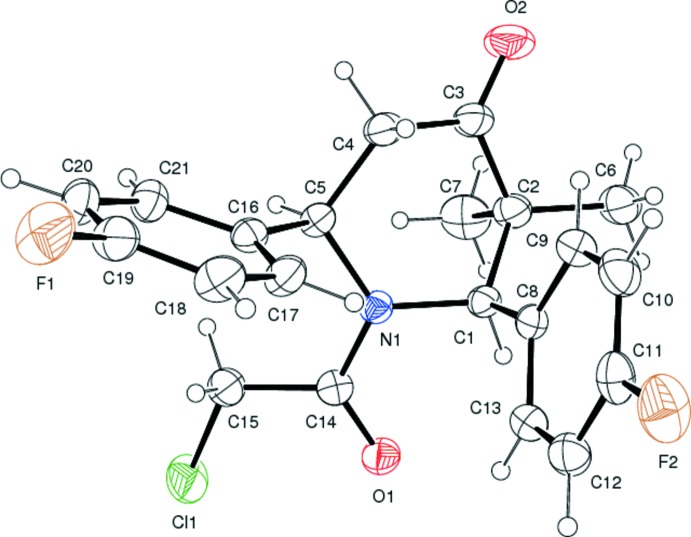
The mol­ecular structure of the title compound, showing 30% probability displacement ellipsoids.

The mol­ecular structure of the title compound is shown in Fig. 1 ▶. The sum of the bond angles around atom N1 (359.6°) confirms *sp* ^2^ hybridization. The N1—C14 [1.356 (2) Å] and C14—O1 [1.221 (2) Å] bond distances indicate the presence electron delocalization in this part of the mol­ecule. The six-membered piperidine ring adopts a slightly distorted boat conformation. The benzene rings form a dihedral angle of 87.43 (1)°. The equatorial and axial orientation of the methyl substituents bonded to atom C2 are described by the N1—C1—C2—C6 and N1—C1—C2—C7 torsion angles of −117.45 (16)° and −57.2 (2)°, respectively. A weak intra­molecular C—H⋯π inter­action is observed, which involves the C8–C13 benzene ring (see Table 1 ▶).

## Supra­molecular features   

**Figure 2:**
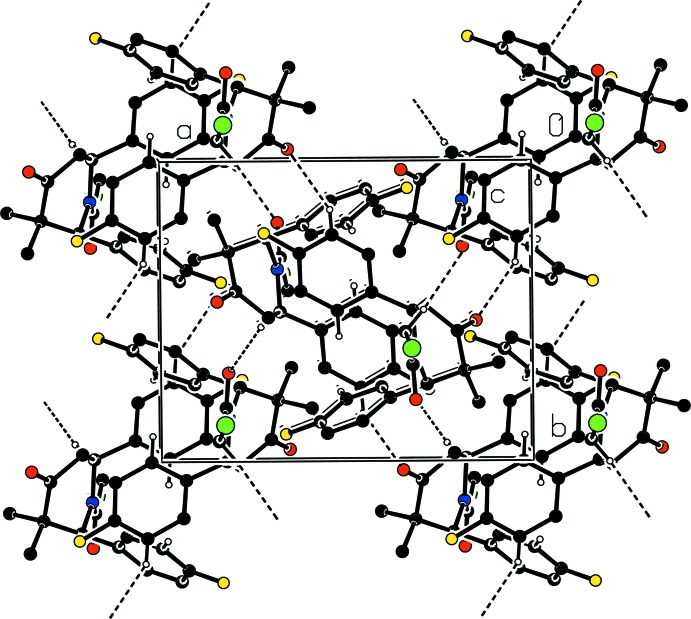
Part of the crystal structure showing weak hydrogen bonds as dashed lines. H atoms not involved in the hydrogen bonds or weak C—H⋯π stacking inter­actions are not shown.

In the crystal, weak C—H⋯O hydrogen bonds and weak C—H⋯π inter­actions link mol­ecules, forming a three-dimensional network (Fig. 2 ▶). Atom O1 acts an acceptor for two weak C—H⋯O hydrogen bonds forming an 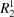
(7) ring.

## Database survey   

A search of the Cambridge Structural Database (Version 5.35, updates to May 2014; Allen, 2002 ▶) revealed four closely related structures in which the dihedral angles between the benzene rings (which are given in square brackets) can be compared to the title compound. These structures are *r*-2,*c*-6-bis­(4-fluoro­phen­yl)-*t*-3,*t*-5-di­methyl­piperidin-4-one [50.4 (1)°] (Gayathri *et al.*, 2008*a* ▶), *r*-2,*c*-6-bis­(4-chloro­phen­yl)-*c*-3,*t*-3-di­methyl­piperidin-4-one [77.23 (7)°] (Llango *et al.*, 2008 ▶), *r*-2,*c*-6-bis­(4-chloro­phen­yl)-*t*-3-isopropyl-1-nitro­sopiperidin-4-one [21.56°] (Gayathri *et al.*, 2008*b* ▶) and *r*-2,*c*-6-bis­(4-chloro­phen­yl)-*t*-3-iso­propyl­piperidin-4-one [52.4 (1)°] (Thiruval­luvar *et al.*, 2007 ▶).

## Synthesis and crystallization   

The synthesis followed the procedure of Aridoss *et al.* (2007 ▶). To a stirred solution of 3,3-dimethyl-2,6-bis­(*p*-fluoro­phen­yl) piperidin-4-one (1.4 g, 5 mmol), and tri­ethyl­amine (2 ml, 14.4 mmol) in benzene (20 ml), di­chloro­acetyl­chloride (1 ml, 10 mmol) in benzene (20 ml) was added dropwise for about half an hour. Stirring was continued with mild heating using a magnetic stirrer for 7 h. The progress of the reaction was monitored by TLC. After the completion of reaction, it was poured into water and extracted with ether. The collected ether extracts were then washed well with 3% sodium bicarbonate solution and dried over anhydrous Na_2_SO_4_. The pasty mass obtained was purified by crystallization from a benzene–petroleum ether solution (333–353 K) in the ratio of 95:5. X-ray quality crystals were grown by slow evaporation of an ethanol solution of the title compound at ambient temperature.

## Refinement   

Crystal data, data collection and structure refinement details are summarized in Table 2 ▶. All H atoms were placed in calculated positions (C—H = 0.93–0.97 Å) and included in the refinement in a riding-model approximation with *U*_iso_(H) = 1.2*U*_eq_(C) or 1.5*U*_eq_(C_meth­yl_).

## Supplementary Material

Crystal structure: contains datablock(s) I. DOI: 10.1107/S1600536814021278/lh5727sup1.cif

Structure factors: contains datablock(s) I. DOI: 10.1107/S1600536814021278/lh5727Isup2.hkl

Supporting information file. DOI: 10.1107/S1600536814021278/lh5727Isup3.tif

Supporting information file. DOI: 10.1107/S1600536814021278/lh5727Isup4.cml

CCDC reference: 1026047

Additional supporting information:  crystallographic information; 3D view; checkCIF report

## Figures and Tables

**Table 1:** Hydrogen-bond geometry (Å, °) *Cg*1 and *Cg*2 are the centroids of the C16–C21 and C8–C13 rings, respectively.

*D*—H⋯*A*	*D*—H	H⋯*A*	*D*⋯*A*	*D*—H⋯*A*
C5—H5⋯O1^i^	0.98	2.50	3.453 (2)	165
C15—H15*A*⋯O1^i^	0.97	2.46	3.429 (3)	174
C20—H20⋯O2^ii^	0.93	2.45	3.298 (3)	151
C10—H10⋯*Cg*1^iii^	0.93	2.66	3.499 (2)	151
C17—H17⋯*Cg*2	0.93	2.85	3.771 (2)	170

Symmetry codes: (i) 
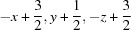
; (ii) 
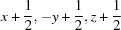
; (iii) 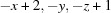.

**Table 2:** Experimental details

Crystal data	
Chemical formula	C_21_H_20_ClF_2_NO_2_
*M*_r_	391.83
Crystal system, space group	Monoclinic, *P*2_1_/*n*
Temperature (K)	293
*a*, *b*, *c* (Å)	13.5270 (3), 10.0150 (2), 15.2560 (3)
β (°)	113.803 (1)
*V* (Å^3^)	1890.97 (7)
*Z*	4
Radiation type	Mo *K*α
μ (mm^−1^)	0.24
Crystal size (mm)	0.35 × 0.30 × 0.30

Data collection	
Diffractometer	Bruker APEXII CCD
Absorption correction	Multi-scan (*SADABS*; Bruker, 2004 ▶)
*T*_min_, *T*_max_	0.914, 0.944
No. of measured, independent and observed [*I* > 2σ(*I*)] reflections	17109, 3327, 2643
*R*_int_	0.030
(sin θ/λ)_max_ (Å^−1^)	0.595

Refinement	
*R*[*F* ^2^ > 2σ(*F* ^2^)], *wR*(*F* ^2^), *S*	0.038, 0.097, 1.02
No. of reflections	3327
No. of parameters	245
H-atom treatment	H-atom parameters constrained
Δρ_max_, Δρ_min_ (e Å^−3^)	0.34, −0.42

Computer programs: *APEX2* and *SAINT* (Bruker, 2004 ▶), *SIR92* (Altomare *et al.*, 1993 ▶), *SHELXL97* (Sheldrick, 2008 ▶), *ORTEP-3 for Windows* (Farrugia, 2012 ▶) and *PLATON* (Spek, 2009 ▶).

## supplementary crystallographic information

### Crystal data

**Table:**

C_21_H_20_ClF_2_NO_2_	*F*(000) = 816
*M**_r_* = 391.83	*D*_x_ = 1.376 Mg m^−^^3^
Monoclinic, *P*2_1_/*n*	Mo *K*α radiation, λ = 0.71073 Å
Hall symbol: -P 2yn	Cell parameters from 6438 reflections
*a* = 13.5270 (3) Å	θ = 2.6–27.4°
*b* = 10.0150 (2) Å	µ = 0.24 mm^−^^1^
*c* = 15.2560 (3) Å	*T* = 293 K
β = 113.803 (1)°	Block, colourless
*V* = 1890.97 (7) Å^3^	0.35 × 0.30 × 0.30 mm
*Z* = 4	

### Data collection

**Table:**

Bruker APEXII CCD diffractometer	3327 independent reflections
Radiation source: fine-focus sealed tube	2643 reflections with *I* > 2σ(*I*)
Graphite monochromator	*R*_int_ = 0.030
ω and φ scans	θ_max_ = 25.0°, θ_min_ = 1.7°
Absorption correction: multi-scan (*SADABS*; Bruker, 2004)	*h* = −15→16
*T*_min_ = 0.914, *T*_max_ = 0.944	*k* = −11→11
17109 measured reflections	*l* = −18→18

### Refinement

**Table:**

Refinement on *F*^2^	Secondary atom site location: difference Fourier map
Least-squares matrix: full	Hydrogen site location: inferred from neighbouring sites
*R*[*F*^2^ > 2σ(*F*^2^)] = 0.038	H-atom parameters constrained
*wR*(*F*^2^) = 0.097	*w* = 1/[σ^2^(*F*_o_^2^) + (0.0329*P*)^2^ + 1.0437*P*] where *P* = (*F*_o_^2^ + 2*F*_c_^2^)/3
*S* = 1.02	(Δ/σ)_max_ = 0.001
3327 reflections	Δρ_max_ = 0.34 e Å^−^^3^
245 parameters	Δρ_min_ = −0.42 e Å^−^^3^
0 restraints	Extinction correction: *SHELXL*, Fc^*^=kFc[1+0.001xFc^2^λ^3^/sin(2θ)]^-1/4^
Primary atom site location: structure-invariant direct methods	Extinction coefficient: 0.0104 (10)

### Special details

**Table:**

Geometry. All e.s.d.'s (except the e.s.d. in the dihedral angle between two l.s. planes) are estimated using the full covariance matrix. The cell e.s.d.'s are taken into account individually in the estimation of e.s.d.'s in distances, angles and torsion angles; correlations between e.s.d.'s in cell parameters are only used when they are defined by crystal symmetry. An approximate (isotropic) treatment of cell e.s.d.'s is used for estimating e.s.d.'s involving l.s. planes.
Refinement. Refinement of *F*^2^ against ALL reflections. The weighted *R*-factor *wR* and goodness of fit *S* are based on *F*^2^, conventional *R*-factors *R* are based on *F*, with *F* set to zero for negative *F*^2^. The threshold expression of *F*^2^ > σ(*F*^2^) is used only for calculating *R*-factors(gt) *etc*. and is not relevant to the choice of reflections for refinement. *R*-factors based on *F*^2^ are statistically about twice as large as those based on *F*, and *R*-factors based on ALL data will be even larger.

### Fractional atomic coordinates and isotropic or equivalent isotropic displacement parameters (Å^2^)

**Table:**

	*x*	*y*	*z*	*U*_iso_*/*U*_eq_	
C1	0.78563 (14)	−0.24331 (17)	0.58593 (12)	0.0380 (4)	
H1	0.7638	−0.3211	0.6129	0.046*	
C2	0.68341 (15)	−0.1993 (2)	0.49931 (14)	0.0480 (5)	
C3	0.70229 (17)	−0.0678 (2)	0.46053 (15)	0.0528 (5)	
C4	0.78997 (17)	0.0206 (2)	0.52812 (14)	0.0495 (5)	
H4A	0.7675	0.1126	0.5120	0.059*	
H4B	0.8542	0.0072	0.5157	0.059*	
C5	0.82206 (14)	0.00286 (17)	0.63608 (13)	0.0395 (4)	
H5	0.7706	0.0536	0.6534	0.047*	
C6	0.64617 (19)	−0.3065 (2)	0.42125 (16)	0.0658 (7)	
H6A	0.6349	−0.3890	0.4479	0.099*	
H6B	0.7004	−0.3188	0.3964	0.099*	
H6C	0.5798	−0.2789	0.3705	0.099*	
C7	0.59232 (17)	−0.1729 (3)	0.53339 (19)	0.0726 (7)	
H7A	0.5767	−0.2537	0.5593	0.109*	
H7B	0.5287	−0.1437	0.4802	0.109*	
H7C	0.6149	−0.1050	0.5819	0.109*	
C8	0.88456 (15)	−0.28756 (17)	0.57008 (12)	0.0379 (4)	
C9	0.90707 (17)	−0.2532 (2)	0.49221 (14)	0.0493 (5)	
H9	0.8574	−0.2026	0.4430	0.059*	
C10	1.00201 (19)	−0.2928 (2)	0.48641 (15)	0.0560 (6)	
H10	1.0167	−0.2686	0.4341	0.067*	
C11	1.07354 (17)	−0.3676 (2)	0.55817 (16)	0.0535 (5)	
C12	1.05468 (17)	−0.4051 (2)	0.63625 (15)	0.0530 (5)	
H12	1.1047	−0.4566	0.6846	0.064*	
C13	0.96006 (15)	−0.36480 (19)	0.64150 (13)	0.0440 (5)	
H13	0.9462	−0.3899	0.6942	0.053*	
C14	0.82422 (14)	−0.17695 (19)	0.74942 (13)	0.0399 (4)	
C15	0.84427 (18)	−0.0661 (2)	0.82215 (14)	0.0524 (5)	
H15A	0.7946	0.0068	0.7929	0.063*	
H15B	0.9172	−0.0325	0.8408	0.063*	
C16	0.93284 (15)	0.06468 (18)	0.68828 (13)	0.0402 (4)	
C17	1.02557 (16)	0.0042 (2)	0.69010 (15)	0.0493 (5)	
H17	1.0217	−0.0819	0.6661	0.059*	
C18	1.12403 (17)	0.0696 (2)	0.72703 (16)	0.0586 (6)	
H18	1.1862	0.0289	0.7278	0.070*	
C19	1.12733 (17)	0.1957 (2)	0.76238 (15)	0.0566 (6)	
C20	1.03892 (18)	0.2570 (2)	0.76548 (15)	0.0554 (6)	
H20	1.0443	0.3414	0.7925	0.066*	
C21	0.94109 (17)	0.1907 (2)	0.72754 (14)	0.0483 (5)	
H21	0.8798	0.2315	0.7284	0.058*	
N1	0.81566 (12)	−0.13914 (14)	0.66131 (10)	0.0366 (4)	
O1	0.81730 (12)	−0.29307 (13)	0.77065 (9)	0.0514 (4)	
O2	0.65146 (16)	−0.03393 (17)	0.37891 (11)	0.0883 (6)	
F1	1.22180 (11)	0.26360 (14)	0.79297 (12)	0.0865 (5)	
F2	1.16548 (11)	−0.40863 (16)	0.55128 (10)	0.0810 (5)	
Cl1	0.82752 (6)	−0.12066 (7)	0.92455 (4)	0.0788 (2)	

### Atomic displacement parameters (Å^2^)

**Table:**

	*U*^11^	*U*^22^	*U*^33^	*U*^12^	*U*^13^	*U*^23^
C1	0.0399 (10)	0.0328 (9)	0.0374 (10)	−0.0039 (8)	0.0116 (8)	0.0023 (7)
C2	0.0406 (11)	0.0451 (11)	0.0462 (11)	−0.0035 (9)	0.0049 (9)	0.0039 (9)
C3	0.0533 (12)	0.0460 (12)	0.0462 (12)	0.0049 (10)	0.0068 (10)	0.0079 (9)
C4	0.0551 (12)	0.0385 (11)	0.0475 (11)	−0.0011 (9)	0.0131 (10)	0.0088 (8)
C5	0.0398 (10)	0.0331 (9)	0.0450 (10)	0.0005 (8)	0.0165 (8)	0.0021 (8)
C6	0.0652 (15)	0.0552 (14)	0.0524 (13)	−0.0129 (11)	−0.0017 (11)	0.0000 (10)
C7	0.0391 (12)	0.0891 (18)	0.0793 (17)	0.0016 (12)	0.0132 (11)	0.0083 (14)
C8	0.0435 (10)	0.0326 (9)	0.0356 (9)	−0.0046 (8)	0.0139 (8)	−0.0030 (7)
C9	0.0580 (13)	0.0489 (12)	0.0391 (11)	0.0019 (10)	0.0177 (10)	0.0035 (9)
C10	0.0683 (14)	0.0617 (14)	0.0473 (12)	−0.0067 (11)	0.0329 (11)	−0.0053 (10)
C11	0.0484 (12)	0.0604 (13)	0.0554 (13)	−0.0021 (10)	0.0248 (10)	−0.0176 (11)
C12	0.0488 (12)	0.0577 (13)	0.0473 (12)	0.0078 (10)	0.0138 (10)	−0.0019 (10)
C13	0.0481 (11)	0.0456 (11)	0.0373 (10)	0.0021 (9)	0.0162 (9)	0.0016 (8)
C14	0.0371 (10)	0.0427 (11)	0.0434 (10)	−0.0002 (8)	0.0199 (8)	0.0021 (8)
C15	0.0667 (14)	0.0494 (12)	0.0476 (12)	0.0031 (10)	0.0299 (11)	0.0005 (9)
C16	0.0444 (11)	0.0374 (10)	0.0389 (10)	−0.0043 (8)	0.0168 (8)	0.0033 (8)
C17	0.0474 (12)	0.0377 (10)	0.0588 (13)	−0.0013 (9)	0.0173 (10)	0.0013 (9)
C18	0.0412 (12)	0.0545 (13)	0.0726 (15)	0.0002 (10)	0.0152 (11)	0.0066 (11)
C19	0.0460 (12)	0.0532 (13)	0.0582 (13)	−0.0159 (10)	0.0084 (10)	0.0044 (10)
C20	0.0652 (14)	0.0450 (12)	0.0548 (13)	−0.0138 (11)	0.0230 (11)	−0.0084 (9)
C21	0.0542 (12)	0.0440 (11)	0.0520 (12)	−0.0063 (9)	0.0268 (10)	−0.0052 (9)
N1	0.0377 (8)	0.0338 (8)	0.0383 (8)	−0.0020 (6)	0.0154 (7)	0.0011 (6)
O1	0.0692 (10)	0.0427 (8)	0.0495 (8)	−0.0042 (7)	0.0313 (7)	0.0050 (6)
O2	0.1047 (14)	0.0628 (11)	0.0543 (10)	−0.0054 (10)	−0.0126 (9)	0.0190 (8)
F1	0.0524 (8)	0.0701 (9)	0.1148 (12)	−0.0234 (7)	0.0107 (8)	−0.0013 (8)
F2	0.0621 (9)	0.1066 (12)	0.0843 (10)	0.0072 (8)	0.0400 (8)	−0.0227 (8)
Cl1	0.1233 (6)	0.0731 (4)	0.0600 (4)	−0.0112 (4)	0.0577 (4)	−0.0075 (3)

### Geometric parameters (Å, º)

**Table:**

C1—N1	1.483 (2)	C10—C11	1.357 (3)
C1—C8	1.519 (3)	C10—H10	0.9300
C1—C2	1.542 (2)	C11—F2	1.353 (2)
C1—H1	0.9800	C11—C12	1.368 (3)
C2—C3	1.507 (3)	C12—C13	1.375 (3)
C2—C6	1.530 (3)	C12—H12	0.9300
C2—C7	1.541 (3)	C13—H13	0.9300
C3—O2	1.203 (2)	C14—O1	1.221 (2)
C3—C4	1.505 (3)	C14—N1	1.356 (2)
C4—C5	1.535 (3)	C14—C15	1.515 (3)
C4—H4A	0.9700	C15—Cl1	1.754 (2)
C4—H4B	0.9700	C15—H15A	0.9700
C5—N1	1.485 (2)	C15—H15B	0.9700
C5—C16	1.517 (2)	C16—C21	1.382 (3)
C5—H5	0.9800	C16—C17	1.383 (3)
C6—H6A	0.9600	C17—C18	1.384 (3)
C6—H6B	0.9600	C17—H17	0.9300
C6—H6C	0.9600	C18—C19	1.367 (3)
C7—H7A	0.9600	C18—H18	0.9300
C7—H7B	0.9600	C19—F1	1.353 (2)
C7—H7C	0.9600	C19—C20	1.362 (3)
C8—C9	1.384 (3)	C20—C21	1.382 (3)
C8—C13	1.389 (3)	C20—H20	0.9300
C9—C10	1.381 (3)	C21—H21	0.9300
C9—H9	0.9300		
			
N1—C1—C8	110.21 (14)	C8—C9—H9	119.5
N1—C1—C2	109.50 (14)	C11—C10—C9	119.07 (19)
C8—C1—C2	119.33 (16)	C11—C10—H10	120.5
N1—C1—H1	105.6	C9—C10—H10	120.5
C8—C1—H1	105.6	F2—C11—C10	118.9 (2)
C2—C1—H1	105.6	F2—C11—C12	119.0 (2)
C3—C2—C6	111.25 (17)	C10—C11—C12	122.1 (2)
C3—C2—C7	105.53 (18)	C11—C12—C13	118.4 (2)
C6—C2—C7	109.02 (18)	C11—C12—H12	120.8
C3—C2—C1	110.54 (15)	C13—C12—H12	120.8
C6—C2—C1	111.49 (16)	C12—C13—C8	121.65 (18)
C7—C2—C1	108.80 (17)	C12—C13—H13	119.2
O2—C3—C4	120.44 (19)	C8—C13—H13	119.2
O2—C3—C2	122.40 (19)	O1—C14—N1	122.96 (17)
C4—C3—C2	117.15 (16)	O1—C14—C15	120.95 (17)
C3—C4—C5	118.07 (17)	N1—C14—C15	116.09 (16)
C3—C4—H4A	107.8	C14—C15—Cl1	112.00 (14)
C5—C4—H4A	107.8	C14—C15—H15A	109.2
C3—C4—H4B	107.8	Cl1—C15—H15A	109.2
C5—C4—H4B	107.8	C14—C15—H15B	109.2
H4A—C4—H4B	107.1	Cl1—C15—H15B	109.2
N1—C5—C16	113.80 (14)	H15A—C15—H15B	107.9
N1—C5—C4	111.60 (15)	C21—C16—C17	118.52 (18)
C16—C5—C4	107.94 (15)	C21—C16—C5	119.36 (17)
N1—C5—H5	107.8	C17—C16—C5	121.83 (17)
C16—C5—H5	107.8	C16—C17—C18	121.11 (19)
C4—C5—H5	107.8	C16—C17—H17	119.4
C2—C6—H6A	109.5	C18—C17—H17	119.4
C2—C6—H6B	109.5	C19—C18—C17	118.1 (2)
H6A—C6—H6B	109.5	C19—C18—H18	121.0
C2—C6—H6C	109.5	C17—C18—H18	121.0
H6A—C6—H6C	109.5	F1—C19—C20	118.8 (2)
H6B—C6—H6C	109.5	F1—C19—C18	118.4 (2)
C2—C7—H7A	109.5	C20—C19—C18	122.8 (2)
C2—C7—H7B	109.5	C19—C20—C21	118.3 (2)
H7A—C7—H7B	109.5	C19—C20—H20	120.8
C2—C7—H7C	109.5	C21—C20—H20	120.8
H7A—C7—H7C	109.5	C20—C21—C16	121.1 (2)
H7B—C7—H7C	109.5	C20—C21—H21	119.4
C9—C8—C13	117.70 (18)	C16—C21—H21	119.4
C9—C8—C1	125.28 (17)	C14—N1—C1	117.35 (14)
C13—C8—C1	116.97 (16)	C14—N1—C5	122.27 (15)
C10—C9—C8	121.09 (19)	C1—N1—C5	119.95 (14)
C10—C9—H9	119.5		
			
N1—C1—C2—C3	58.3 (2)	C1—C8—C13—C12	−177.07 (17)
C8—C1—C2—C3	−70.0 (2)	O1—C14—C15—Cl1	−12.3 (2)
N1—C1—C2—C6	−177.45 (16)	N1—C14—C15—Cl1	168.29 (14)
C8—C1—C2—C6	54.3 (2)	N1—C5—C16—C21	−135.35 (17)
N1—C1—C2—C7	−57.2 (2)	C4—C5—C16—C21	100.2 (2)
C8—C1—C2—C7	174.54 (17)	N1—C5—C16—C17	50.9 (2)
C6—C2—C3—O2	31.2 (3)	C4—C5—C16—C17	−73.6 (2)
C7—C2—C3—O2	−86.9 (3)	C21—C16—C17—C18	−2.4 (3)
C1—C2—C3—O2	155.6 (2)	C5—C16—C17—C18	171.43 (18)
C6—C2—C3—C4	−147.7 (2)	C16—C17—C18—C19	0.4 (3)
C7—C2—C3—C4	94.2 (2)	C17—C18—C19—F1	−175.83 (19)
C1—C2—C3—C4	−23.3 (3)	C17—C18—C19—C20	2.2 (3)
O2—C3—C4—C5	157.1 (2)	F1—C19—C20—C21	175.26 (19)
C2—C3—C4—C5	−23.9 (3)	C18—C19—C20—C21	−2.8 (3)
C3—C4—C5—N1	35.1 (2)	C19—C20—C21—C16	0.7 (3)
C3—C4—C5—C16	160.89 (18)	C17—C16—C21—C20	1.8 (3)
N1—C1—C8—C9	−105.5 (2)	C5—C16—C21—C20	−172.16 (18)
C2—C1—C8—C9	22.4 (3)	O1—C14—N1—C1	6.3 (3)
N1—C1—C8—C13	72.05 (19)	C15—C14—N1—C1	−174.27 (16)
C2—C1—C8—C13	−160.01 (16)	O1—C14—N1—C5	178.74 (17)
C13—C8—C9—C10	−0.9 (3)	C15—C14—N1—C5	−1.8 (2)
C1—C8—C9—C10	176.67 (18)	C8—C1—N1—C14	−103.14 (17)
C8—C9—C10—C11	0.6 (3)	C2—C1—N1—C14	123.70 (17)
C9—C10—C11—F2	178.63 (18)	C8—C1—N1—C5	84.23 (18)
C9—C10—C11—C12	0.0 (3)	C2—C1—N1—C5	−48.9 (2)
F2—C11—C12—C13	−178.82 (18)	C16—C5—N1—C14	67.7 (2)
C10—C11—C12—C13	−0.2 (3)	C4—C5—N1—C14	−169.80 (16)
C11—C12—C13—C8	−0.2 (3)	C16—C5—N1—C1	−120.01 (17)
C9—C8—C13—C12	0.7 (3)	C4—C5—N1—C1	2.4 (2)

### Hydrogen-bond geometry (Å, º)

Cg1 and Cg2 are the centroids of the C16–C21 and C8–C13 rings, respectively.

**Table:**

*D*—H···*A*	*D*—H	H···*A*	*D*···*A*	*D*—H···*A*
C5—H5···O1^i^	0.98	2.50	3.453 (2)	165
C15—H15*A*···O1^i^	0.97	2.46	3.429 (3)	174
C20—H20···O2^ii^	0.93	2.45	3.298 (3)	151
C10—H10···*Cg*1^iii^	0.93	2.66	3.499 (2)	151
C17—H17···*Cg*2	0.93	2.85	3.771 (2)	170

Symmetry codes: (i) −*x*+3/2, *y*+1/2, −*z*+3/2; (ii) *x*+1/2, −*y*+1/2, *z*+1/2; (iii) −*x*+2, −*y*, −*z*+1.
